# Chemical Characterization of Mediterranean Macroalgae With a Focus on Antioxidant Molecules Through the Use of Liquid Chromatographic Techniques

**DOI:** 10.1002/jssc.70373

**Published:** 2026-02-11

**Authors:** Federica Vento, Emanuela Trovato, Francesca Rigano, Giuseppe Micalizzi, Daniele Giuffrida, Luigi Mondello

**Affiliations:** ^1^ Department of Chemical Biological, Pharmaceutical and Environmental Sciences Messina Institute of Technology University of Messina Messina Italy; ^2^ Department of Biomedical Dental, Morphological and Functional Imaging Sciences University of Messina Messina Italy; ^3^ Department of Chemical Biological, Pharmaceutical and Environmental Sciences Chromaleont S.R.L. University of Messina Messina Italy

**Keywords:** antioxidants, greenness assessment, microconstituents, seaweeds, vitamins

## Abstract

Seaweeds represent an excellent vegetable protein resource capable of supporting the objectives of the sustainable blue economy. Today, attention toward algae is growing, due to their applications, both in biofuel production and as an alternative source of healthy food and nutraceuticals. The objective of this work was to valorize marine macroalgae coming from the Mediterranean area in order to be able to expand their potential use in the nutraceutical and pharmaceutical industries, maintaining the circularity of financial and biological resources. Micronutrients with higher antioxidant activity were determined in 10 samples, specifically four red algae, four brown algae, and two green algae. In particular, vitamin C was analyzed using a reversed phase (RP)‐HPLC system coupled to photodiode array (PDA) detection, following extraction with an acidified aqueous solution. Vitamin E analysis was performed using a normal‐phase HPLC system, following extraction with n‐hexane and taking advantage of the high selectivity and sensitivity of fluorescence detector. Vitamin B12 was extracted with an acidified solution of methanol and water and analyzed by RP‐HPLC system coupled to PDA and MS, the latter operated under selected ion monitoring mode to increase instrumental sensitivity and selectivity. Finally, carotenoids and pigments were extracted with an acetone/methanol solvent mixture and analyzed by RP‐HPLC system coupled to PDA and MS to exploit the complementarity between MS and UV spectra for identification purposes. Overall, the validated HPLC methods confirmed the presence of vitamin E in all the samples analyzed, with highest levels obtained in two brown algae, namely, *Undaria pinnatifida* (1.54 ± 0.08 mg/kg) and *Himanthalia elongata* (0.93 ± 0.02 mg/kg), whereas vitamins C and B12 were detected only in two macroalgae species, including the widely consumed *Porphyra* sp., commercially known as Nori. Finally, carotenoids were mainly determined in the largely consumed *U. pinnatifida* sample, commercially known as Wakame.

## 1 Introduction

Global population growth, resource depletion, and climate change require the adoption of new and sustainable methods to feed a rapidly growing world population. A successful strategy could consist of the use of widely available biomasses until now only marginally exploited, as represented by the seas and oceans, which, according to the biomass report of the Joint Research Center (last update 2022), are only marginally used despite constituting over 70% of the earth's surface [1]. In the same year, the European Commission released the Communication *Towards a Strong and Sustainable EU Algae Sector* to highlight the “role of algae as an important source of alternative protein for a sustainable food system” [2]. In this regard, the consumption of algae aligns with most of the 17 objectives of the well‐known Agenda 2030 promoted by United Nations, being widely accessible, nutritious, and characterized by a low environmental footprint, as many algae grow wild without the need for additional resources (freshwater or arable land) and efficiently capture CO_2_ while producing high biomass yields. Despite the strong flavor of algae, consumer acceptance is generally positive compared to other novel food sources, such as insects or cultured meat [3]. Moreover, in addition to direct consumption, algae have been extensively studied for various biological and biomedical purposes [4] and can be used to produce food supplements, such as dietary fiber extract, algal oil rich in omega‐3 (ω‐3) fatty acids, and oleoresin rich in antioxidants, such as carotenoids [5]. Some of these products have been already included in the novel food catalogue [6], after a thorough risk‐assessment by EFSA's scientific panel, which evaluates detailed data on composition, toxicity, allergenicity, production process, and expected intake to ensure consumer safety. For this reason, there is growing interest in the comprehensive chemical characterization of algae [7].

As a whole food, algae have regularly been used by the East Asian population (Japan, Korea, China) since ancient times, with positive health effects such as decrease in obesity, diabetes, heart disease, and cancer, as well as increased longevity [8]. The macrobiotic diet, which arrived in Europe from Japan, contributed to the introduction of algae into western diet. *Undaria pinnatifida* and *Laminaria digitata* are the brown algae most used in the macrobiotic diet. Their chemical characterization highlighted a well‐balanced composition in terms of macronutrients (proteins, lipids, carbohydrates, polysaccharides, essential amino acids, essential fatty acids, and dietary fiber) and high levels of antioxidant molecules such as carotenoids and phenolics, minerals (iodine, iron, potassium, zinc, copper, magnesium, and calcium), and vitamins, both water and fat soluble [9, 10, 11, 12, 13, 14]. Kolb and co‐workers in 2004 pointed out that the vitamin content in algae is 10–20 times higher than in land plants [15]. Moreover, regarding their vitamin composition, it should be underlined that vitamin B12 is mainly found in animal‐derived foods; therefore, the presence of cobalamin compounds in some algae represents a noteworthy advantage compared to most plant‐based foods and may contribute to reducing the risk of deficiency in individuals following a vegan diet, although their bioavailability can vary among species [11, 14, 16, 17]. In the present work, the content of vitamin E, vitamin C, and vitamin B12 with high antioxidant power was investigated in 10 samples of macroalgae. Specifically, four samples of red algae (*Chondrus crispus*, *Palmaria palmata*, *Porphyra* sp., and *Gracilaria gracilis*), four brown algae (*Laminaria digitata*, *Saccharina latissima*, *Himanthalia elongata*, and *Undaria pinnatifida*), and two green algae (*Ulva lactuca*) were analyzed. This research includes the elucidation of the carotenoid and pigment composition as microconstituents responsible for the classification of algae according to pigmentation [5]. The obtained results are important to expand the knowledge about composition of macroalgae and their potential use in food, nutraceutical, and pharmaceutical industries, thus maintaining the circularity of financial and biological resources. From an analytical point of view, all methods applied to vitamin determination were fully validated in terms of linearity, limit of detection (LOD), limit of quantification (LOQ), accuracy, and intra‐ and inter‐day precision, aiming at providing a guideline for the characterization of such components in macroalgae.

## 2 Materials and Methods

### 2.1 Chemical and Reagents

HPLC purity grade methanol (MeOH), water, *n*‐hexane (Hex), 2‐propanol (IPA), methyl *tert*‐butyl ether (MTBE), ACS reagent grade acetic acid, acetone, sodium chloride (NaCl), formic acid, *m*‐phosphoric acid, certified reference material of ascorbic acid (vitamin C) and cyanocobalamin (vitamin B12), α‐tocopherol (purity grade ≥ 95.5%), β‐tocopherol (purity grade ≥ 99.2%), γ‐tocopherol (purity grade ≥ 96%), δ‐tocopherol (purity grade ≥ 90%), and α‐tocotrienol (purity grade ≥ 97%) were purchased from Merck Life Science (Merck KGaA Darmstadt, Germany).

### 2.2 Samples

Commercial edible seaweeds were purchased from an Italian company and were labelled as originating from the northern Adriatic Sea. In detail, the macroalgae were classified as follows: three red algae (*Chondrus crispus*, *Palmaria palmata, Porpyra* sp.), four brown algae (*Laminaria digitata, Saccharina latissima, Himanthalia elongata*, and *Undaria pinnatifida*), and one green algae (*Ulva lactuca*). Furthermore, two wild macroalgae (*Ulva lactuca* and *Gracilaria gracilis*) were collected from the coastal lagoon of the northern Adriatic Sea. Sampling sites were selected based on the presence of Gracilaria spp. and the occurrence of algal infestations.

The sampling was conducted onshore (on land) and offshore (open sea). The wild algae were collected, respectively, in two specific periods, May–June and September–October, when algal blooms usually occur. The complete list of analyzed macroalgae, including scientific name, common name, *phylum*, and class, is reported in Table 1.

**TABLE 1:** List of analyzed algae, along with their scientific names, common names, phylum, and class.

#	Scientific name	Common name	Phylum	Class
1	*Undaria pinnatifida*	Wakame	Ochrophyta (brown)	Pheophyceae
2	*Laminaria digitata*	Kombu	Ochrophyta (brown)	Pheophyceae
3	*Himanthalia elongata*	Sea spaghetti	Ochrophyta (brown)	Pheophyceae
4	*Saccharina latissima*	Saccharina	Ochrophyta (brown)	Pheophyceae
5	*Porphyra* sp.	Nori	Rhodophyta (red)	Bangiophyceae
6	*Chondrus crispus*	Carragheen	Rhodophyta (red)	Florideophyceae
7	*Palmaria palmata*	Dulse	Rhodophyta (red)	Florideophyceae
8	*Ulva lactuca*	Sea lettuce	Chlorophyta (green)	Ulvophyceae
9	*Ulva lactuca* ^a^	Sea lettuce^a^	Chlorophyta (green)	Ulvophyceae
10	*Gracilaria gracilis* ^a^	*Gracilaria* ^a^	Rhodophyta (red)	Florideophyceae

^a^ Wild algae.

### 2.3 Analysis of Vitamin E

For the absolute quantification of vitamin E, 0.5 g of sample was weighted and extracted with 2.5 mL of MeOH solution of ascorbic acid (0.01%, v/v) and 5 mL of Hex. After placing the sample under constant agitation for 30 min, 2.5 mL of aqueous solution of NaCl (1%, v/v) was added. The mixture was centrifuged by using the Neya‐16R centrifuge. The upper Hex phase was collected, and the residue was re‐extracted twice with 5 mL of Hex. The collected Hex phases were pooled, and the solvent was dried under nitrogen stream. The residue was re‐dissolved in 250 µL of Hex and injected into the HPLC system. All operations were conducted in the dark and minimized impact on the air. The analyses were carried out using a HPLC Nexera‐X2 system (Shimadzu, Duisburg, Germany), equipped with a fluorescence detector (FLD). Tocopherols were separated on an Ascentis Si column (250 × 4.6 mm, L. × i.d., 5 µm particle size) (Merck Life Science), operated in isocratic mode with Hex/IPA (99:1, v/v) mobile phase at a flow rate of 1.7 mL/min (run time 7 min). Oven temperature was set at 25°C. Samples were analyzed in triplicate and the injection volume was 20 µL. Detector wavelength was 290 nm for excitation and 330 nm for emission. Data acquisition was performed using the LabSolution software (ver. 5.97, Shimadzu). Quantitative analysis was carried out by external calibration, and the method was validated in terms of linearity, LOD, LOQ, precision, and accuracy, according to Eurachem guidelines [18]. Calibration curves were built in the concentration range 0.1–25 mg/L (five replicates for six concentration levels). Precision was measured at the lowest concentration, both at intra‐ and inter‐day level. Accuracy (R%) was assessed by adding known amount of α‐tocopherol and γ‐tocopherol (the only detected vitamers in the analyzed algae) to 0.5 g of algae at three concentration levels (0.1, 1, and 25 mg/L) and applying Equation (1), viz. comparing the signal (in terms of area) differences between spiked and unspiked samples with the signal obtained for the pure standard at the same concentration level of the spike:

(1) $$R=\frac{\mathrm{Are}a_{\mathrm{sample}\;\mathrm{spiked}}-\;\mathrm{Are}a_{\mathrm{sample}\;\mathrm{unspiked}}}{\mathrm{Are}a_{\mathrm{standard}}}\;$$

Detection and quantification limits were calculated by adding 25 µL of α‐tocopherol and γ‐tocopherol (0.1 mg/L) to 0.5 g of a blank sample (the extract residue was used), extracted according to the procedure abovementioned, and the analysis were repeated 9 times. Equations (2) and (3) were applied for calculating the LOD and LOQ, respectively:

(2) $$LOD=3\times s_{0}^{\prime }$$

(3) $$LOQ=10\times s_{0}^{\prime },$$

where $s_{0}^{\prime }=\frac{Dev.std}{\sqrt{9}}$

### 2.4 Analysis of Vitamin C

For absolute quantification of vitamin C, 1.5 g of sample was weighed and extracted with 10 mL of aqueous solution of *m*‐phosphoric acid (4.5%, v/v). After that, the sample was subjected to a centrifuge at 21°C for 15 min at 1000 rpm and at 4500 rpm for additional 2 min. Subsequently, the liquid phase was collected, and the residue was re‐extracted twice. Then, the extracts were collected in a volumetric flask of 25 mL and brought to volume with aqueous solution of *m*‐phosphoric acid (4.5%, v/v). All operations were conducted in the dark and minimized impact with the air, as the molecule under examination degrades in the presence of light and air. Finally, 2 mL of the extract was taken and filtered with a 25 mm, nylon 66, 0.45‐µm syringe filter (BGB Analytik AG, Böckten, Switzerland) and injected into the HPLC [19]. Vitamin C analysis was conducted using an HPLC Nexera‐X2 system (Shimadzu), coupled to an SPD‐M30A photodiode array (PDA) detector. Chromatographic separation was performed by using an Ascent Express C18 (15 cm × 4.6 mm, 2.7 µm) column (Merck Life Science) and an aqueous solution of acetic acid (0.1% v/v, pH 3) as mobile phase at a flow of 0.7 mL/min in isocratic elution mode. The injection volume was 20 µL. The oven temperature was set at 30°C. UV–Vis spectra were acquired in the range 200–500 nm, and vitamin C was detected at 245 nm, with a sampling frequency of 12.5 Hz. The absolute quantification of the target compound in commercial and infesting seaweed was performed according to standard addition calibration method to limit the matrix effect of such complex samples. Calibration curves were built in the range 0–50 mg/L. The method was validated in terms of linearity, LOD, LOQ, precision, and accuracy (at two concentration levels, 10 and 50 mg/L) according to Eurachem guidelines [18] and equations reported in Section 2.3. Data were acquired and processed using Labsolution software (ver. 5.97, Shimadzu).

### 2.5 Analysis of Vitamin B12

For the absolute quantification of vitamin B12, 0.3 g of sample was weighed and extracted with 6 mL of a solution of methanol and ultrapure water (25:75%, v/v), then formic acid was added to obtain a pH of 4. The extraction process was conducted in the dark since vitamin B12 is light sensitive. The mixture was centrifuged at 550 rpm for 30 min; afterward, the solution was centrifugated at 21°C for 10 min at 4500 rpm by using the same centrifuge described in Section 2.3. The supernatant was collected and 450 µL was brought to a final volume of 500 µL with the solution used for the extraction. Finally, a volume of 5 µL of sample was injected into a HPLC Nexera‐X2 system (Shimadzu), coupled to a single‐quadrupole LCMS‐2020 mass spectrometer equipped with an electrospray (ESI) interface (Shimadzu). Chromatographic separation was performed by using an Ascentis Express C18 (10 cm × 2.1 mm, 2.7 µm) column (Merck Life Science). A gradient of aqueous solution of formic acid (2%, v:v) (A) and IPA solution of formic acid (2%, v:v) (B) was applied at a mobile phase flow rate of 0.4 mL/min: 0–5 min, 0%–35% B; 5–7 min, 35%–100% B (held for 1 min). The oven temperature was set at 35°C. MS conditions were as follows: full scan acquisition, both ESI positive (+) and negative (−) in the *m/z* range 150–1500; selected ion monitoring (SIM) acquisition in (+) polarity of ions at *m/z* 1355 ([M]^+^), *m/z* 678 ([M+H]^2+^), and the characteristic fragments at *m/z* 997 (loss of the cobalt‐bound nucleotide ligand, consisting of 5,6‐dimethylbenzimidazole linked to the ribose‐phosphate group), *m/z* 1209 (loss of the nucleobase 5,6‐dimethylbenzimidazole), *m/z* 359 (ion corresponding to the nucleotide ligand of vitamin B12, consisting of 5,6‐dimethylbenzimidazole linked to the ribose‐phosphate group); nebulizing and drying gas flows (N_2_) were 1.5 L/min and 15 L/min, respectively; event time: 0.2 s; DL temperature: 250°C; heat block: 200°C. The absolute quantification of the target compound in commercial and infesting seaweed was performed according to the standard addition calibration method in the range 0–8 mg/L, integrating SIM chromatogram corresponding to *m/z* 678, which provided the highest signal. The method was validated in terms of linearity, LOD, LOQ, precision, and accuracy (at two concentration levels, 1 and 8 mg/L) according to Eurachem guidelines [18] and equations reported in Section 2.3. The data were acquired and processed using Labsolution software (ver. 5.97, Shimadzu).

### 2.6 Analysis of Carotenoids and Pigment

For the analysis of the carotenoid and pigment, 100 mg of the sample was weighed and extracted with 1.5 mL of acetone/methanol solution (1:1, v/v). Then the mixture was sonicated for 20 min and the supernatant was collected. The extraction was repeated twice, and the three extracts were combined and dried under a stream of nitrogen. Finally, the dry extracts were dissolved in 750 µL of a mixture of methanol/MTBE (1:1, v: v) and filtered with a 25 mm, nylon 66, 0.45 µm syringe filter prior to HPLC analysis (BGB Analytik AG) [20]. Carotenoid and pigment analyses were carried out by using an HPLC Nexera‐X2 system (Shimadzu), coupled to an SPD‐M30A PDA detector serially connected to the LCMS‐2020 (Shimadzu) detector, equipped with an atmospheric pressure chemical ionization (APCI) interface. Separation was carried out on an Ascentis express C30 column 250 × 4.6 mm, 5 µm, (Merck Life Science). The mobile phases consisted of MeOH/MTBE/water (90:8:2, v/v/v; eluent A) and MeOH/MTBE/water, (8:90:2, v/v/v; eluent B). The following gradient program was used: 0–20 min, 0–20% B; 20–60 min, 20–50% B, 60–65 min, 50–100% B (held for 2 min); finally, the system was brought to its initial condition and reconditioned for 13 min. The flow rate was 0.8 mL/min, and the injection volume was set at 20 µL. The oven temperature was set at 30°C. UV–Vis spectra were acquired in the range of 220–700 nm, and chromatograms were extracted at 450 nm (sampling frequency: 4.1667 Hz). MS conditions were as follows: full scan acquisition, both APCI positive (+) and negative (−) in the *m/z* range: 200–950; nebulizing gas flow (N_2_): 4 L/min; event time: 0.5 s; interface temperature: 350°C; DL temperature: 300°C; heat block: 300°C. Data processing and handling were performed using Labsolution software (ver. 5.97, Shimadzu).

## 3 Results and Discussion

### 3.1 Vitamin E Content

A cold method was chosen for the extraction of vitamin E. In detail, MeOH was used to break the plant cell walls of algae, and Hex was chosen due to its affinity with fat‐soluble molecules and compatibility with the employed NP‐HPLC method based on an isocratic elution at 99% of Hex. In comparison with the extraction method reported in the literature [21], sample amount and reagent volume were proportionally reduced (halved). Moreover, butylated hydroxytoluene, normally used as antioxidant molecule to preserve the content of tocopherols during the extraction but highly toxic for aquatic life with long‐lasting effects, was replaced by the not hazardous ascorbic acid. These slight modifications had a positive effect on the overall greenness of the method, despite further improvements are necessary to reduce waste generation and the amounts of toxic solvents employed (see detailed discussion in Section 3.5). Regarding identified and quantified tocopherols, only α and γ vitamers were detected, while β and δ vitamers and α‐tocotrienol were not detected in any sample under investigation. In detail, significant amounts of α‐tocopherol were found in most samples, with the highest amounts detected in two brown commercial algae, namely, *Undaria pinnatifida* (1.54 ± 0.08 mg/kg) and *Himanthalia elongata* (0.84 ± 0.01 mg/kg), followed by the wild seaweeds *Ulva lactuca* (0.60 ± 0.10 mg/kg) and *Gracilaria gracilis* (0.35 ± 0.01 mg/kg). A recent review paper [11] summarized the content of vitamins in seaweeds of different geographical origins, and it is possible to note that the content of vitamin E in the wild seaweeds *Ulva lactuca* analyzed in the present work was comparable to that reported for samples collected in the Black Sea and markedly higher than that reported for samples from the Ireland coast. Based on the same review paper [11], the vitamin E content in the *Himanthalia elongata* seaweed here investigated was of the same order of magnitude as that reported for samples from Spanish coast. It is noteworthy as the other commercial samples have a significantly lower content of vitamin E, that is, in the range of 0.05–0.19 mg/kg, highlighting possible deterioration during storage and placement on the market (e.g., following drying process). On the other hand, wild macroalgae were freeze‐dried in the lab immediately after their collection. Low amounts of γ‐tocopherol were quantified in only two commercial samples: *Himanthalia elongata* (0.09 ± 0.01 mg/kg) and *Ulva lactuca* (0.05 ± 0.01 mg/kg). As an illustrative case study, the chromatogram of *Himanthalia elongata* extract, compared to the standard mixture chromatographic profile, is depicted in Figure S1. Quantitative results are shown in Table 2, along with the figures of merits. In detail, high sensitivity and good linearity were achieved, as expected for the sensitive and selective FLD detector, with a slope of the linear regression equation of 3E+06 and coefficients of determination (*R* ^2^) of 0.9998 for both vitamers. The recovery values obtained were in a range between 80 and 120%, except for γ‐tocopherol at the highest concentration level, which is significantly higher than the concentration range observed in the sample. Moreover, intra‐ and inter‐day precision were below 5%, highlighting the high repeatability of the method.

**TABLE 2:** Vitamin E content in the algae analyzed, along with figures of merits (linearity, limits of detection [LOD], and limit of quantification [LOQ]), intra‐ and inter‐day repeatability (expressed as CV %), and accuracy (expressed as percentage values).

		α‐Tocopherol (mg/kg)	γ‐Tocopherol (mg/kg)
**LOD**		0.05	0.02
**LOQ**		0.15	0.06
**Precision intra‐day %**		1.34	4.34
**Precision inter‐day %**		1.33	4.3
**Accuracy %**	0.1 mg/L	112.9	119.9
	1 mg/L	88.6	80.8
	25 mg/L	118.0	68.3
**Samples**	*Undaria pinnatifida*	1.54 ± 0.08	< LOD
	*Laminaria digitata*	0.19 ± 0.01	< LOD
	*Himanthalia elongata*	0.84 ± 0.01	0.09 ± 0.01
	*Saccharina latissima*	LOD	< LOD
	*Porphyra* sp.	0.28 ± 0.01	< LOD
	*Chondrus crispus*	LOD	< LOD
	*Palmaria palmata*	LOD	< LOD
	*Ulva lactuca*	LOD	LOQ
	*Ulva lactuca* ^a^	0.60 ± 0.10	< LOD
	*Gracilaria gracilis* ^a^	0.35 ± 0.01	< LOD

^a^ Wild algae.

### 3.2 Vitamin C Content

Vitamin C is a water‐soluble vitamin, and its content in algae is related to a multitude of factors, such as genetic, geographical, and processing‐related factors. Consequently, evaluating and generalizing its levels across different species is challenging. Moreover, vitamin C degrades easily and is present in traces in algae, as confirmed by data reported in literature [22]. Therefore, the entire extraction process must be carried out protected from light and heat sources. Furthermore, an acid pH stabilizes the molecule during both the extraction process and chromatographic separation [19]. In this regard, following a survey of literature papers on the determination of vitamin C in different vegetable matrices, several procedures were evaluated, differing for extraction media and treatment (e.g., centrifugation and vortex, only centrifugation, only vortex, and purification using polyvinylpolypyrrolidone). The analyses of the extracts obtained following different extraction procedures highlighted that centrifugation at controlled temperature (21°C) is the preferred operation to promote the solid–liquid extraction limiting as possible degradation issues. Moreover, different acids were used as extraction media, namely, formic acid (0.5%, v:v) [23], oxalic acid (1%, v:v) [24], mixture of acetic (8%, v:v), and *m*‐phosphoric acid (3%, w:v) [25], and *m*‐phosphoric acid (4.5%, w:v) [19]. The latter resulted in the most effective and selective for the determination of vitamin C through an RP‐HPLC‐PDA method under isocratic elution. Indeed, vitamin C eluted at 2.5 min and appeared baseline separated from other matrix components, differently from other extraction procedures, which resulted in low intensity signal (poor extraction recovery) and/or coelution with other matrix constituents (poor selectivity of extraction process). Nonetheless, considering the high complexity of samples, the absolute quantification of the target compound was carried out through the standard addition calibration method to fully mitigate potential matrix effects. As an example, the chromatogram of the Himanthalia elongata extract is shown in Figure S2, along with the chromatogram of pure vitamin C.

Overall, fast analysis time, simple and widely accessible instrumental setup, use of water as main extraction medium, and HPLC eluent make the employed method quite green and practical (see detailed discussion in Section 3.5).

Regarding the quantitative results, among all the analyzed samples, vitamin C was identified only in the brown alga *Himanthalia elongata* (72.9 mg/kg) and in the red alga *Porphyra* sp. (20.2 mg/kg), a brown and a red alga, respectively. Table 3 reports such quantitative results along with the results of method validation. LOD and LOQ values were in the range 0.004–0.08 mg/kg, low enough for the determination of such microconstituents at ppb levels. Intra‐ and inter‐day precision were both below 0.35%, highlighting the high repeatability of the method. Accuracy was measured at two concentration levels, and the obtained results were in the range of 100%–120%, pointing out the efficient recovery of the analyte.

**TABLE 3:** Vitamin C content in the analyzed algae along with linear regression equation, coefficient of determination (*R* ^2^ as indicator of linearity), limit of detection (LOD) and limit of quantification (LOQ), intra‐ and inter‐day repeatability (expressed as CV %), and accuracy (expressed as percentage values).

Species	Vitamin C (mg/kg)	Linear regression equation	*R* ^2^	LOD (mg/kg)	LOQ (mg/kg)	Precision intra‐day %	Precision inter‐day %	Accuracy % 10 mg/L	Accuracy % 50 mg/L
*Undaria pinnatifida*	< LOD	—	—	0.004	0.01	—	—	—	—
*Laminaria digitata*	< LOD	—	—	0.01	0.03	—	—	—	—
*Himanthalia elongata*	72.9	*y* = (109,963 ± 1435)*x* + 481,006 ± 33,772	0.9958	0.02	0.08	0.34	0.32	118.4	116.4
*Saccharina latissima*	< LOD	—	—	0.01	0.02	—	—	—	—
*Porphyra* sp.	20.2	*y* = (94,161 ± 373)*x* + 113,977 ± 9202	0.9996	0.02	0.05	0.14	0.18	117.2	100.1
*Chondrus crispus*	< LOD	—	—	0.01	0.05	—	—	—	—
*Palmaria palmata*	< LOD	—	—	0.01	0.03	—	—	—	—
*Ulva lactuca*	< LOD	—	—	0.005	0.02	—	—	—	—
*Ulva lactuca* ^a^	< LOD	—	—	0.02	0.06	—	—	—	—
*Gracilaria gracilis* ^a^	< LOD	—	—	0.01	0.03	—	—	—	—

^a^ Wild algae.

### 3.3 Vitamin B12 Content

Similar to vitamin C, vitamin B12 belongs to the class of water‐soluble vitamins, and its content in algae widely varies depending on genetic and growth conditions [26]. Moreover, it is photosensitive and present in seaweeds at trace levels, typically in the order of a few µg per 100 g of dry weight [27]; therefore, its analytical determination can be challenging. The extraction was performed in the dark according to the procedure optimized by Susanti and co‐workers [28], proportionally miniaturized (1:10) to reduce solvent consumption and waste generation, according to green analytical chemistry principles [29]. To further increase the greenness of the analytical method, compared to the chromatographic conditions used by Susanti et al. [28], methanol in the mobile phase was replaced by 2‐propanol, which is a recommended solvent according to an overall ranking of solvents aligning different classification methods elaborated by several pharmaceutical companies and institutions [30]. Moreover, rather than using an advanced and more expensive HPLC‐MS/MS system, a simpler and more accessible HPLC‐MS instrumentation was used, thus enhancing the practicality or blueness of the analytical method (see detailed discussion in Section 3.5). To increase instrumental sensitivity and reduce LOD and LOQ values, the analyses were carried out in SIM mode through the monitoring of characteristic fragments of the vitamin B12 (see Section 2.5 for the list of monitored fragments ions). Nonetheless, the absolute quantification of the target compound was carried out through the standard addition calibration method due to the high complexity of the sample, that is, the presence of interfering matrix constituents responsible for possible suppression/enhancement ionization effects in the MS source. The SIM chromatogram of Laminaria digitata extract for the ion at *m/z* 678 is reported in Figure S3, in comparison with the analysis of pure vitamin B12. Moreover, SIM chromatograms at *m/z* 997 and *m/z* 1209 were also included as confirmatory ions for the identification of vitamin B12 in real samples.

Vitamin B12 was detected only in *Laminaria digitata* (96.4 µg/100 g) and *Porphyra* sp. (39.6 µg/100 g), a brown and a red alga, respectively, as reported in Table 4 along with the figures of merit obtained for the validated method. Specifically, LOD and LOQ were in the range of nanograms for 100 g of dried alga, intra‐ and inter‐day precision were below 4%, and accuracy ranged between 74% and 120%.

**TABLE 4:** Vitamin B12 content in the analyzed algae along with linear regression equation, coefficient of determination (*R* ^2^ as indicator of linearity), limit of detection (LOD) and limit of quantification (LOQ), intra‐ and inter‐day repeatability (expressed as CV %), and accuracy (expressed as percentage values).

Species	Vitamin B12 (µg/100 g)	Linear regression equation	*R* ^2^	LOD (µg/100 g)	LOQ (µg/100 g)	Precision Intra‐day %	Precision Inter‐day %	Accuracy % 1 mg/L	Accuracy % 8 mg/L
*Undaria pinnatifida*	< LOD	—	—	0.000	0.002	—	—	—	—
*Laminaria digitata*	96.4	*y* = (227,066 ± 3095)*x* + 131,351 ± 11,666	0.9970	0.001	0.003	0.80	0.85	119.80	74.32
*Himanthalia elongata*	< LOD	—	—	0.000	0.001	—	—	—	—
*Saccharina latissima*	< LOD	—	—	0.000	0.001	—	—	—	—
*Porphyra* sp.	39.6	*y* = (149,564 ± 2841)*x* + 35,572 ± 10,711	0.9943	0.003	0.010	3.87	3.96	118.98	94.50
*Chondrus crispus*	< LOD	—	—	0.001	0.002	—	—	—	—
*Palmaria palmata*	< LOD	—	—	0.001	0.002	—	—	—	—
*Ulva lactuca*	< LOD	—	—	0.000	0.001	—	—	—	—
*Ulva lactuca* ^a^	< LOD	—	—	0.000	0.001	—	—	—	—
*Gracilaria gracilis* ^a^	< LOD	—	—	0.000	0.001	—	—	—	—

^a^ Wild algae.

In the specific case of vitamin B12, concentrations reported in the literature show wide variability, with non‐detectable levels in most cases, including *Laminaria digitata* [11]. In contrast, different *Porphyra* species have been reported to contain significant amounts of vitamin B12 [14], consistent with the results of the present study.

### 3.4 Carotenoids and Pigments

Carotenoids are oil‐soluble unsaturated molecules found in plants, mushrooms, algae, and photosynthetic bacteria. Many studies have shown the beneficial effects of carotenoids in preventing aging and aging‐related diseases thanks to their antioxidant activity, that is, the ability to contrast oxidative stress and reactive oxygen species (ROS) implicated in cardiovascular, neurodegenerative, liver, skin, and eye diseases [31]. Also, their therapeutic potential has been widely investigated and recently reviewed by Ortega‐Regules et al. [32], who pinpointed their role toward metabolic syndrome, such as hypertension, obesity, and atherogenic dyslipidemia. Fucoxanthin, lutein, and zeaxanthin were cited among the most biologically active carotenoids, and brown algae were identified as the main natural source of fucoxanthin.

**FIGURE 1:**
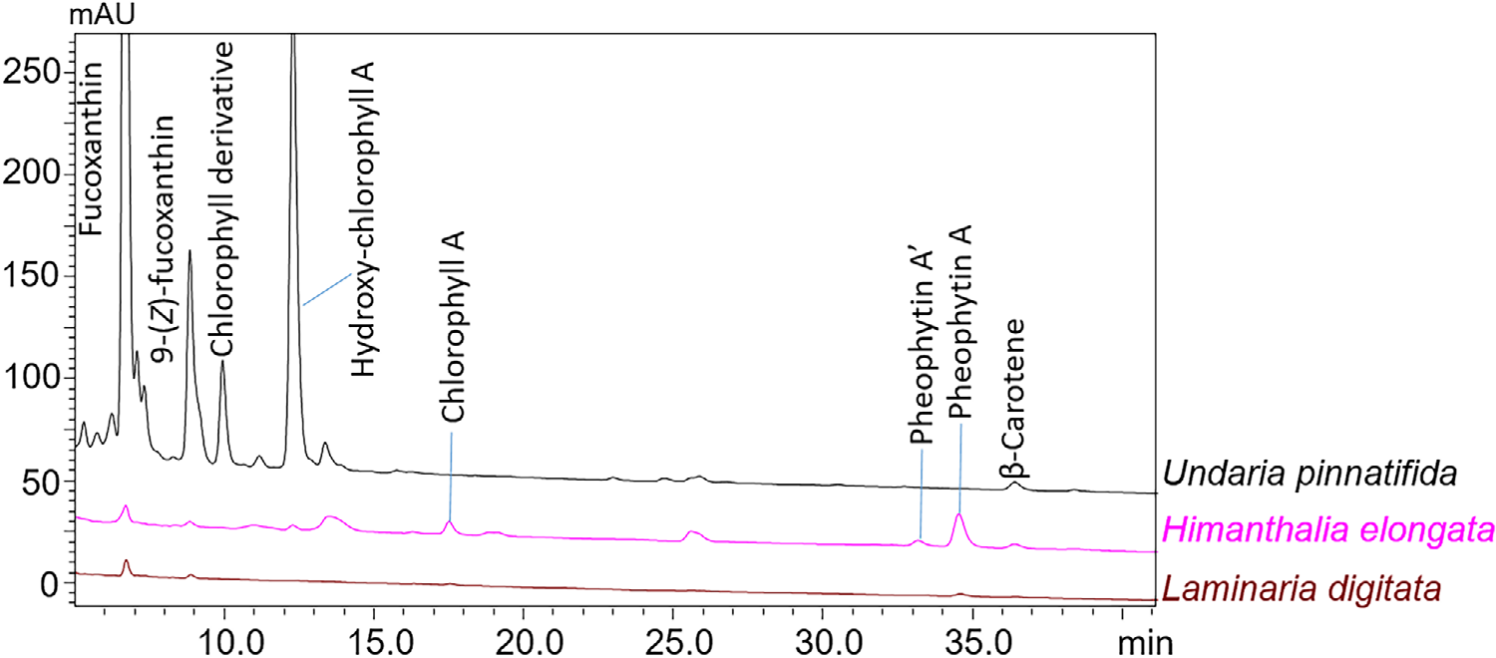
Comparison of the HPLC‐PDA chromatographic profiles (λ = 450 nm) obtained for brown algae *Undaria pinnatifida*, *Himanthalia elongata*, and *Lamindaria digitata*.

The list of carotenoids identified in the samples analyzed in the present work is reported in Table 5, along with MS and UV signals used for their identification and confirmed by literature data [33, 34, 35], which support the discrimination between isomeric compounds through different UV spectra. Particularly, Z‐isomers showed a UV absorption band at lower wavelengths, corresponding to a hypsochromic (blue) shift with respect to the E‐isomer. In detail, according to literature [5, 36], fucoxanthin was confirmed as the main carotenoid in brown algae with *Undaria pinnatifida* showing the most intense signal, significantly higher (> 500 mAU) compared to *Himanthalia elongata* and *Laminaria digitata* (both < 10 mAU). Moreover, 9‐(*Z*) isomer of fucoxanthin was also detected in all the above‐mentioned brown algae, with a stronger signal in *Undaria pinnatifida* (Figure 1 and Table 5).

**TABLE 5:** List of carotenoids detected in the analyzed seaweed samples, along with MS fragments in positive (+) and negative (−) polarity, maximum UV wavelength (*λ*_max_).

Carotenoids and pigments	MS fragments	*λ*_max_ (nm)	Sample
Fucoxanthin	641 (+)^a^ 581 (+)^b^ 411 (+) 658 (−)	446^b^ 468	*Undaria pinnatifida* ^***^, *Himanthalia elongata* ^*^, and *Laminaria digitata* ^*^
9‐(*Z*)‐Fucoxanthin	641 (+)^a^ 581 (+) 658 (−)	442^a^ 461	*Undaria pinnatifida* ^**^, *Himanthalia elongata* ^*^, and *Laminaria digitata* ^*^
Lutein	551 (+)^c^ 568 (−)^c^	421^b^ 443^b^ 472^b^	*Ulva lactuca* ^d^ *^,^* ^**^, *Ulva lactuca* ^*^ *, Porphyra* sp.^*^, and *Gracilaria gracilis* ^d^ *^,^* ^*^
Zeaxanthin	569 (+)^b^ 551 (+)^b^ 568 (−)^c^	422^b^ 449^b^ 477^b^	*Gracilaria gracilis* ^d^ *^,^* ^*^
9‐(*Z*)‐Lutein	551 (+) 568 (−)	421^b^ 438^b^ 466^b^	*Ulva lactuca* ^d^ *^,^* ^*^
β‐Carotene	537 (+)^a)^, ^b)^ 536 (−)^b)^, ^c)^	428^b^ 452^b^ 477^b^	*Undaria pinnatifida* ^*^ and *Himanthalia elongata* ^*^

^a)^ [33].

^b)^ [34].

^c)^ [35].

^d)^Wild algae.

* Signal < 100 mAU

** signal range 100–500 mAU

*** signal > 500 mAU.

**FIGURE 2:**
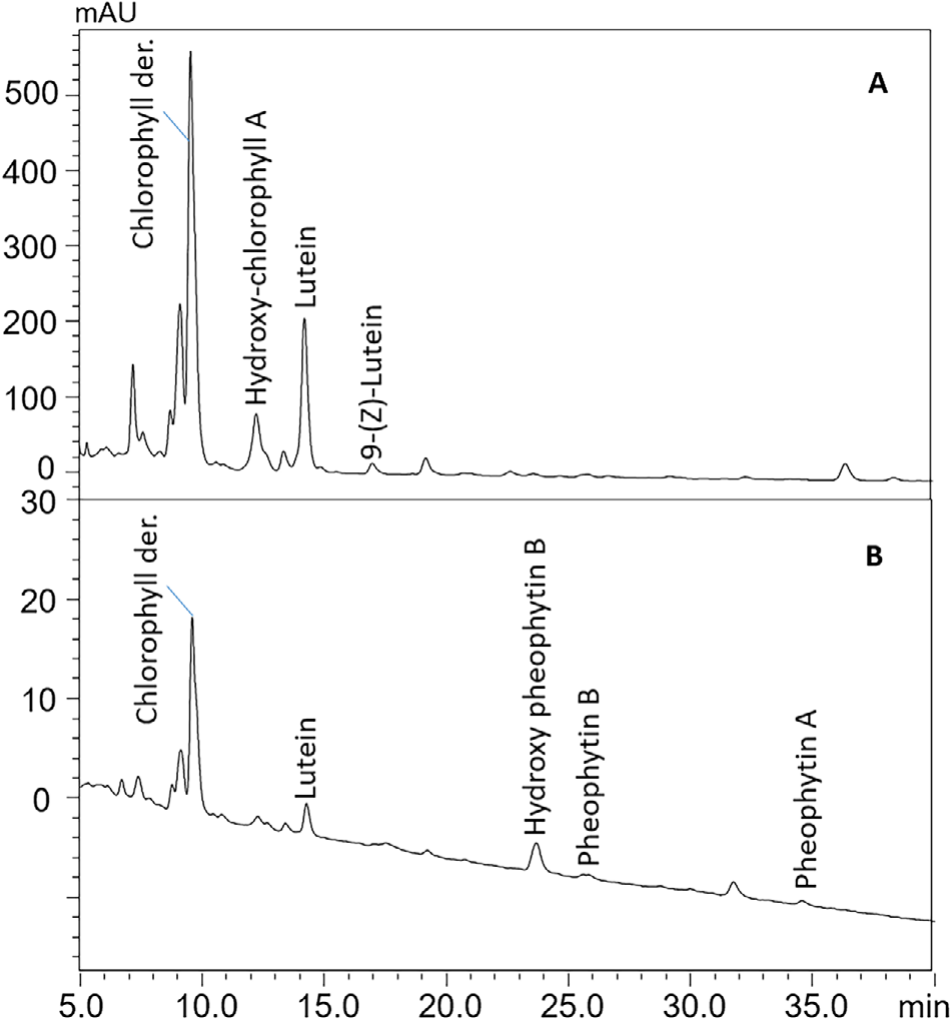
Comparison of the HPLC‐PDA chromatographic profiles (λ = 450 nm) obtained for the (A) wild *Ulva lactuca* and (B) commercial *Ulva Lactuca*.

As for green algae, the wild sample showed an intense signal for lutein in combination with a low signal of the 9‐(*Z*) isomer, while the commercial sample showed only a weak signal for lutein, highlighting as in the case of vitamin E (Section 3.1) a possible deterioration during storage and placement on the market (e.g., following drying process). In fact, wild macroalgae were immediately freeze‐dried in the lab after collection, thus better preserving their carotenoid profile. As tentative confirmation of such a hypothesis, the chromatogram of the wild *Ulva lactuca* sample showed a chlorophyll derivative and hydroxy‐chlorophyll A as main pigments, while the commercial *Ulva Lactuca* sample pointed out the presence of chlorophyll degradation products, namely, pheophytin and hydroxy‐pheophytin. The chromatograms in Figure 2 clearly pinpoint the different carotenoid and pigment composition of wild and commercial *Ulva lactuca* samples.

Finally, carotenoids were detected only in two of the four red algae analyzed, namely, *Gracilaria gracilis* and *Porphyra* sp. Both showed the presence of lutein, while zeaxanthin was identified only in *Gracilaria gracilis*.

Chlorophylls and their derivatives, as essential photosynthetic pigments for plant organisms, were detected in all samples with higher amounts in the green algae samples (Figure 2), followed by the brown alga *Undaria pinnatifida* and the red algae *Gracilaria gracilis* and *Porphyra* sp., in accordance with literature information [5, 17]. The other commercial samples exhibited stronger signals for the degradation products, the pheophytins.

### 3.5 Greenness and Blueness Evaluation

The last step of the present research work consisted of the evaluation of greenness and blueness of the employed analytical methods to verify the alignment with principles of sustainability in laboratory practices [37]. The method for the elucidation of carotenoid and pigment profiles has not been subjected to calculation since no modifications were made to either the sample preparation or the chromatographic separation with respect to a previously published method [20].

**FIGURE 3:**
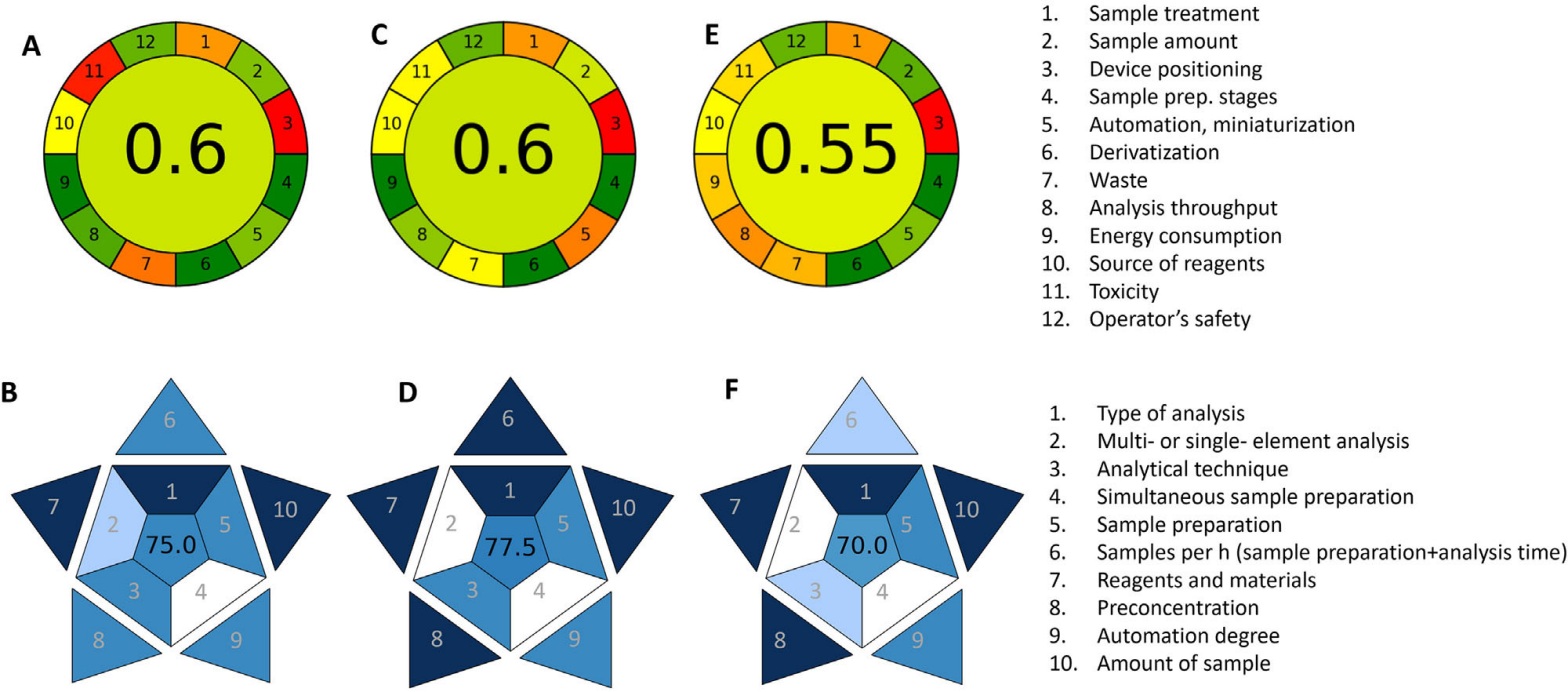
AGREE and BAGI results for the analysis of (A and B) vitamin E, (C and D) vitamin C, and (E and F) vitamin B12.

Regarding the determination of vitamins E, C, and B12, the freely accessible online BAGI (Blue Applicability Grade Index) [38] tool was used for the calculation of the blueness score, and the AGREE [39] calculator was applied for the estimation of the greenness (Figure 3).

As mentioned in Section 3.1, the overall greenness of the method employed for the determination of vitamin E was slightly enhanced compared to more conventional procedures. The removal of the toxic butylated hydroxytoluene increased the score for point 12, while the reduction of sample and reagents amount led to a higher score mainly for points 2 and 5. A slight improvement was also achieved for points 7 (score 0.23 rather than 0.17) and 11 (score 0.07 rather than 0), but future efforts are encouraged to address these aspects. As for practicality, a score of 75.0 was achieved, demonstrating the suitability of the method for routine analysis.

Similar results were achieved for the quantitative analysis of vitamin C, for which the reduced analysis time and the absence of a preconcentration step in the extraction procedure made the method more practical compared to vitamin E (BAGI score 77.5 vs. 75.0).

Lastly, Figure 3E,F shows the pictograms relative to greenness and blueness score assigned to the determination of vitamin B12, highlighting the benefits arising from the reduced amount of sample (points 2, 5 and 7 of AGREE) and toxic reagents (point 11). As in the case of vitamin E, further efforts should be addressed to the removal of toxic reagents. For instance, methanol was replaced with 2‐propanol in the mobile phase composition, but it is still used for the sample preparation. The need for an MS detector operated in SIM mode to detect the analyte, normally present at very low concentrations, is the main factor responsible for the lower AGREE (point 9, related to energy consumption) and BAGI scores compared with vitamins E and C.

## 4 Conclusion

This research work aimed at the development and validation of robust and affordable analytical methods for the determination of antioxidant molecules and vitamin B12 in macroalgae collected in the Mediterranean Sea. The use of simple and relatively cost‐effective analytical procedures was highlighted through the application of the BAGI tool for the estimation of practicality (score ≥ 70 for all methods), as well as the analytical greenness was evaluated, pointing out a quite green score (≥ 0.55) compared to more conventional methods employing larger amount of sample and solvents and/or toxic reagents.

The validated HPLC methods confirmed the presence of vitamin E in all the samples analyzed, whereas vitamins C and B12 were detected only in two macroalgae species, including the widely consumed *Porphyra* sp., commercially known as Nori.

Finally, carotenoids, widely known as important antioxidant constituents, were mainly determined in the largely consumed *Undaria pinnatifida* sample, commercially known as Wakame, and in the wild sample of *Ulva Lactuca*. Hence, the present study intended to extend the knowledge of the composition of wild and commercial macroalgae to investigate their potential applications in food, nutraceutical, and pharmaceutical industries, thereby supporting the circular use of financial and biological resources.

## Author Contributions

**Federica Vento**: investigation, writing – original draft, data curation, validation, visualization. **Emanuela Trovato**: data curation, validation, visualization. **Francesca Rigano**: writing – review and editing, data curation, supervision, conceptualization. **Giuseppe Micalizzi**: conceptualization, writing – review and editing. **Daniele Giuffrida**: data curation, validation. **Luigi Mondello**: supervision, funding acquisition, project administration.

## Conflicts of Interest

The authors declare no conflicts of interest.

## Supporting information




**Supporting File 1**: jssc70373‐sup‐0001‐SuppMat.pdf.

## Data Availability

The data supporting this study's findings are available from the corresponding author upon reasonable request.
