# Supplementary material for: Chemical Characterization of Mediterranean Macroalgae With a Focus on Antioxidant Molecules Through the Use of Liquid Chromatographic Techniques
Source: J Sep Sci. 2026 Feb 11;49(2):e70373. doi: 10.1002/jssc.70373 (PMC12895137; doi:10.1002/jssc.70373)
Supplement: Supplementary file 1 — Supporting File 1: jssc70373‐sup‐0001‐SuppMat.pdf. [file JSSC-49-e70373-s001.pdf]

Chemical characterization of Mediterranean macroalgae with a focus on antioxidant molecules through the use of liquid chromatographic techniques.

Federica Vento[a], Emanuela Trovato[a], Francesca Rigano[a]*, Giuseppe Micalizzi[a], Daniele Giuffrida[b], Luigi Mondello[a,c]

[a]Messina Institute of Technology c/o Department of Chemical, Biological, Pharmaceutical and Environmental Sciences, University of Messina, Viale G. Palatucci 13, 98168 – Messina, Italy

[b]Department of Biomedical, Dental, Morphological and Functional Imaging Sciences, University of Messina, Via Consolare Valeria, 98125 – Messina, Italy

[c]Chromaleont s.r.l., c/o Department of Chemical, Biological, Pharmaceutical and Environmental Sciences, University of Messina Viale G. Palatucci 13, 98168 – Messina, Italy

Corresponding author: Dr. Francesca Rigano, Messina Institute of Technology c/o Department of Chemical, Biological, Pharmaceutical, and Environmental Sciences, University of Messina, Viale G. Palatucci 13, 98168 – Messina, Italy. Email: frigano@unime.it.

Table of contents

Figure S1. HPLC-FLD chromatogram (λ_{ex} 290 nm, λ_{em} 330 nm) of Himanthalia elongata vitamin E extract (black trace) compared to the standard mixture chromatographic profile (pink trace).

Figure S2. HPLC-PDA chromatogram (λ 245 nm) of Himanthalia elongata vitamin C extract (black trace) compared to the analysis of pure vitamin C (pink trace).

Figure S3. SIM chromatogram of Laminaria digitata extract for the ions at *m/z* 678 (black trace), *m/z* 997 and *m/z* 1209, compared to the SIM chromatogram of pure vitamin B12 at *m/z* 678 (pink trace).

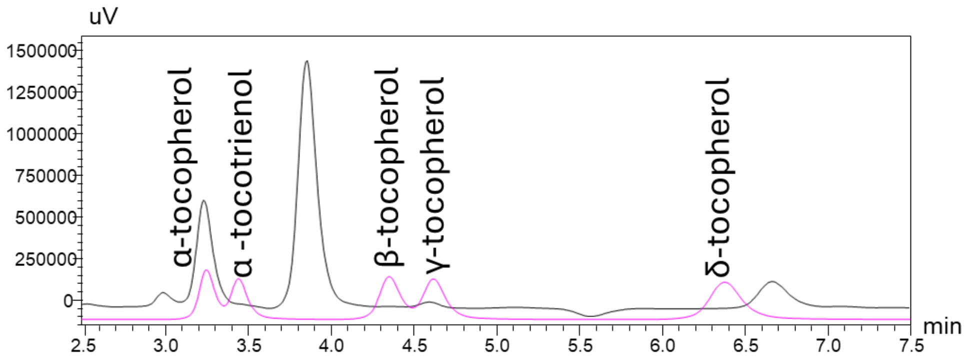

Figure S1. HPLC-FLD chromatogram (λ_{ex} 290 nm, λ_{em} 330 nm) of Himanthalia elongata vitamin E extract (black trace) compared to the standard mixture chromatographic profile (pink trace).

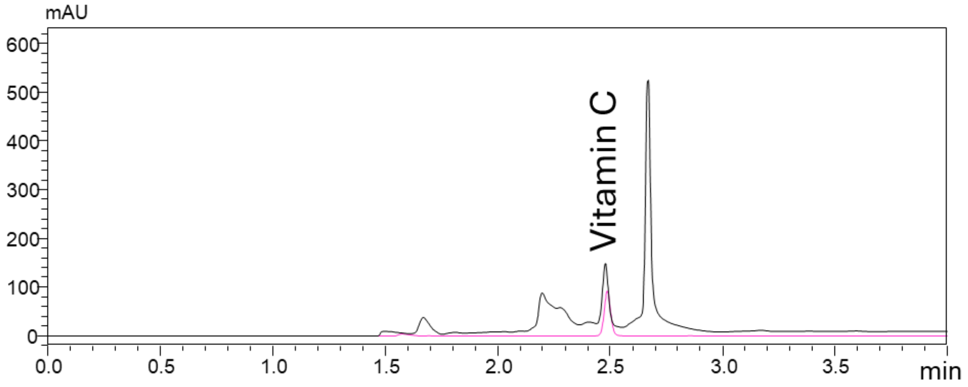

Figure S2. HPLC-PDA chromatogram (λ 245 nm) of Himanthalia elongata vitamin C extract (black trace) compared to the analysis of pure vitamin C (pink trace).

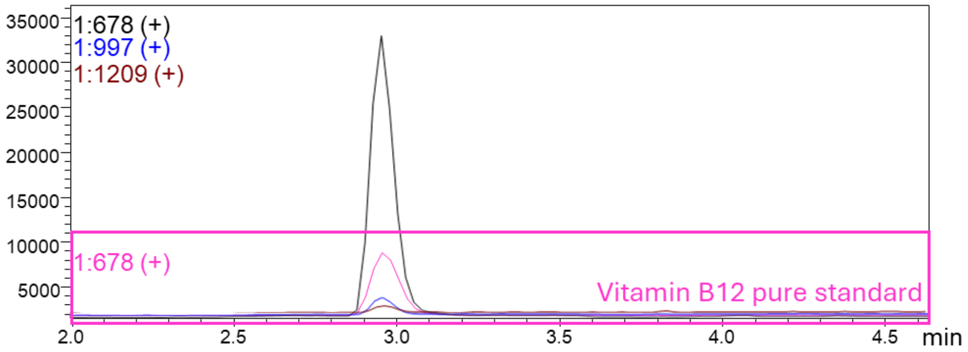

Figure S3. SIM chromatogram of Laminaria digitata extract for the ions at *m/z* 678 (black trace), *m/z* 997 and *m/z* 1209, compared to the SIM chromatogram of pure vitamin B12 at *m/z* 678 (pink trace).
